# HIF-1α/PPAR-γ/SIRT1 axis drives astrocyte senescence and regulates endothelial function via GDNF/NRTN in diabetic retinopathy

**DOI:** 10.1371/journal.pone.0362127

**Published:** 2026-11-06

**Authors:** Jing Wang, Shufen Fang, Ming Sun, Jue Wang, Yongqing Liu, Xuan Wang, Weiwei Liu, Lei Gao

**Affiliations:** 1 Department of Ophthalmology, Shandong Second Provincial General Hospital, Jinan, Shandong, P. R. China; 2 Department of Ophthalmology, Jinan Maternity and Child Care Hospital, Jinan, Shandong, P. R. China; 3 Nova Southeastern University College of Optometry, Davie, Florida, United States of America; 4 Jinan Second People Hospital, Jinan, Shandong, P. R. China; Tokai University School of Medicine: Tokai Daigaku Igakubu Daigakuin Igaku Kenkyuka, JAPAN

## Abstract

**Background:**

Diabetic retinopathy (DR) is a leading cause of blindness in working-age populations. Astrocyte senescence is highly prevalent in DR, but the underlying regulatory mechanism remains unclear. This study aims to investigate the role of the HIF-1α/PPAR-γ/SIRT1 axis in regulating retinal astrocyte senescence and endothelial function in DR.

**Methods:**

Mouse retinal astrocytes were exposed to high glucose (30 mM) to mimic diabetic conditions. Senescence was evaluated by β-galactosidase staining. Signaling pathways were manipulated using HIF-1α inhibitor (PX-478), PPAR-γ inhibitor (T0070907), SIRT1 agonist (SRT2104), and autophagy inhibitor (3-MA). Protein and mRNA levels of key factors were detected by Western blot and RT-qPCR. Astrocytes and retinal microvascular endothelial cells were co-cultured in Transwell systems to assess paracrine regulation on endothelial migration, proliferation, and apoptosis.

**Results:**

High glucose induced astrocyte senescence, inflammatory activation, and downregulation of GDNF and NRTN, accompanied by elevated HIF-1α and PPAR-γ, and reduced SIRT1 expression and autophagy activity. Inhibiting HIF-1α or activating SIRT1 attenuated senescence, apoptosis, and inflammation, restored autophagy, and upregulated GDNF/NRTN secretion. HIF-1α negatively regulated SIRT1 expression via PPAR-γ. Senescent astrocytes impaired endothelial function through reduced GDNF/NRTN; overexpression of GDNF or NRTN in astrocytes preserved endothelial migration and proliferation, and inhibited apoptosis.

**Conclusion:**

HIF-1α/PPAR-γ/SIRT1 axis mediates high glucose-induced astrocyte senescence via autophagy dysfunction. Senescent astrocytes disrupt retinal endothelial homeostasis by reducing GDNF/NRTN secretion. Targeting this axis may represent a novel neurovascular protective therapeutic strategy for DR.

## 1. Introduction

Diabetic retinopathy (DR) is one of the most severe microvascular complications of diabetes mellitus and a major cause of visual impairment and irreversible blindness in adults worldwide [1,2]. The pathogenesis of DR involves a complex cascade of metabolic disorders, oxidative stress, chronic low-grade inflammation, retinal neuroglial damage, microvascular dysfunction, and pathological angiogenesis [3,4]. Increasing evidence indicates that retinal neurodegeneration occurs early in DR, often preceding clinically detectable vascular lesions and representing a critical early target for intervention [5,6].

Astrocytes are the most abundant glial cells in the retina and are essential for maintaining retinal homeostasis, supporting neurons, and stabilizing the blood-retinal barrier (BRB) [7,8]. Recent studies reported that the majority of retinal astrocytes undergo senescence in DR, a proportion significantly higher than that observed in vascular endothelial cells or pericytes [9]. Astrocyte senescence is characterized by irreversible cell cycle arrest, elevated senescence markers, pro-inflammatory senescence-associated secretory phenotype, and impaired neurotrophic support [10]. Accumulating senescent astrocytes contribute to BRB breakdown, vascular hyperpermeability, endothelial dysfunction, and sustained progression of DR [11]. However, the molecular mechanism controlling astrocyte senescence in DR remains poorly defined.

Hypoxia-inducible factor-1α (HIF-1α) is a master transcription factor that responds to hypoxia and metabolic stress [12,13]. Under diabetic conditions, HIF-1α is stabilized and upregulated in the retina, promoting pathological angiogenesis, inflammation, and vascular leakage [14]. It has been documented that the crosstalk between inflammatory signaling and metabolic stress pathways may function in retinal vascular cells [15]. Peroxisome proliferator-activated receptor-γ (PPAR-γ) is a nuclear receptor involved in metabolic regulation, inflammation and cell senescence [16]. Sirtuin 1 (SIRT1) is an NAD^+^-dependent deacetylase that regulates cellular senescence, autophagy, and endothelial function [17]. Emerging evidence has revealed extensive crosstalk among HIF-1α, PPAR-γ, and SIRT1 in metabolic diseases, aging, and tissue injury [18,19]. However, whether these molecules form a functional signaling axis to regulate astrocyte senescence in DR has not been established.

Astrocytes secrete multiple neurotrophic factors including glial cell-derived neurotrophic factor (GDNF) and neurturin (NRTN), which support endothelial cell survival, maintain vascular stability, and protect BRB integrity [20,21]. We hypothesized that high glucose (HG) triggers imbalance of the HIF-1α/PPAR-γ/SIRT1 axis, which suppresses autophagy and promotes astrocyte senescence, leading to reduced GDNF/NRTN secretion and subsequent retinal endothelial dysfunction. This study was designed to verify this hypothesis using in vitro cellular models and to provide mechanistic insights and potential therapeutic targets for DR.

While our previous study [22] demonstrated that pharmacological inhibition of HIF-1α alleviates retinal pathological changes in a DR mouse model, the underlying molecular mechanisms—particularly how HIF-1α regulates astrocyte senescence and glia-vascular crosstalk—remained unexplored. Here, we for the first time establish a functional HIF-1α/PPAR-γ/SIRT1 signaling axis that suppresses autophagy and drives astrocyte senescence under high glucose conditions, and we further reveal that senescent astrocytes impair endothelial function through reduced secretion of GDNF and NRTN. These findings provide a novel mechanistic framework linking astrocytic senescence to endothelial dysfunction in early DR, extending our previous in vivo observations to a detailed cellular and molecular level.

## 2. Materials and methods

### 2.1. Cell culture and treatment

The mouse retinal astrocytes and mouse retinal microvascular endothelial cells required for this study were all purchased from Wuhan Procell Life Science & Technology Co., Ltd. Astrocytes were routinely isolated from 1–3 days old Kunming mice, and endothelial cells from 6–8 weeks old Kunming mice. High glucose (HG, 30 mM) was used to simulate diabetic conditions. Cells were divided into control, HG, HG + PX-478 (HIF-1α inhibitor, 20 μM, MCE, HY-10231) [23], HG + T0070907 (PPAR-γ inhibitor, 20 μM, MCE, HY-13202)(https://www.medchemexpress.cn/mce_publications/11877444.html), HG + DMOG (HIF-1α agonist, 2.5 mM, MCE, HY-15893) [24], HG + SRT2104 (SIRT1 agonist, 3 μM, MCE, HY-15262) [25], and HG + PX-478/SRT2104 + 3-MA (autophagy inhibitor, 5 mM, MCE, HY-19312) [26] groups. All treatments were performed for 24 h except where otherwise noted, and cells were harvested at the indicated time points for downstream assays.

### 2.2. Apoptosis analysis by flow cytometry

Cell apoptosis was evaluated using an Annexin V-FITC/PI apoptosis detection kit. After treatment, cells were harvested, washed with cold PBS, and resuspended in 1 × binding buffer. Then, 5 μL of Annexin V-FITC and 5 μL of propidium iodide (PI) were added, and cells were incubated in the dark for 15 min at room temperature. Apoptosis was analyzed using a flow cytometer, and the apoptosis rate was calculated as the percentage of early and late apoptotic cells.

### 2.3. Enzyme-linked immunosorbent assay (ELISA)

Cell culture supernatants were collected and centrifuged to remove debris. The concentrations of COX-2, IL-6, and IL-8 were measured using commercial ELISA kits following the manufacturer’s protocols. The absorbance at 450 nm was measured using a microplate reader, and cytokine concentrations were calculated according to standard curves.

### 2.4. Western blot analysis

Total protein was extracted from cells using RIPA lysis buffer containing protease and phosphatase inhibitors. Protein concentration was determined using a BCA protein assay kit. Equal amounts of protein (30 μg per lane) were separated by 10–12% SDS-PAGE and transferred to polyvinylidene difluoride (PVDF) membranes. Membranes were blocked with 5% non-fat milk for 1 h at room temperature and then incubated overnight at 4 °C with primary antibodies against p16 (Abcam, ab211542, 1:1000), p21 (Abclonal, A19094, 1:1000), HIF-1α (CST, 36169, 1:1000), PPAR-γ (CST, 2435, 1:1000), SIRT1 (CST, 9475, 1:1000), Beclin 1 (Abclonal, A21191, 1:1000), p62 (CST,23214, 1:1000), LC3B (Abclonal, A19665, 1:1000), GDNF (Proteintech, 26179–1-AP, 1:1000), NRTN (Proteintech, 19709–1-AP, 1:1000), and β-actin (Abclonal, AC026, 1:5000). After washing, membranes were incubated with horseradish peroxidase (HRP)-conjugated secondary antibodies for 1 h at room temperature. Protein bands were visualized using an enhanced chemiluminescence (ECL) detection system and analyzed using ImageJ software, with β-actin as the loading control.

### 2.5. Quantitative real-time PCR (RT-qPCR)

Total RNA was extracted from astrocytes using TRIzol reagent according to the manufacturer’s instructions. RNA concentration and purity were measured using a NanoDrop spectrophotometer. Total RNA (1 μg) was reverse-transcribed into cDNA using a reverse transcription kit. qPCR was performed using a SYBR Green PCR Master Mix on a real-time PCR system. The relative mRNA expression levels were calculated using the 2^−ΔΔCt^ method with β-actin as the internal reference. The primer sequences for target genes including p16, p21, HIF-1α, PPAR-γ, SIRT1, GDNF, NRTN, and VEGF were commercially synthesized. The primer sequences were presented as follows:

GDNF: F: 5’-ACACTCGAGGAGGAAGGACA-3’,
R: 5’-CGGCAGTCCCTTGAAGACAT-3’;
NRTN: F: 5’-CTTTTGCCATCACTTAGAGGCG-3’,
R: 5’-CAAACTTCTTGCCGAGCGAC-3’;
SIRT1: F: 5’-CTGTTTCCTGTGGGATACCTG-3’,
R: 5’-TCCACTGCACAGGCACATAC-3’;
HIF-1α: F: 5’-ACTGTGGACTTGGGGATCAG-3’,
R: 5’-ACTGCCCCCAAGAAGTTGTC-3’;
PPAR-γ: F: 5’-AAGGCTGCAGCGCTAAATTC-3’,
R: 5’-GCAAGGCACTTCTGAAACCG-3’;
β-actin: F: 5’-GGCTGTATTCCCCTCCATCG-3’,
R: 5’-CCAGTTGGTAACAATGCCATGT-3’.

### 2.6. Senescence-associated β-galactosidase staining

Cellular senescence was detected using a senescence-associated β-galactosidase (SA-β-gal) staining kit (Beyotime, China). After treatment, cells were fixed with 4% paraformaldehyde for 15 min at room temperature, washed twice with phosphate-buffered saline (PBS), and incubated with fresh SA-β-gal staining solution at pH 6.0 overnight at 37 °C without CO_2_. Stained cells were observed and imaged under an inverted light microscope. The percentage of SA-β-gal-positive cells was quantified from five random fields per group.

### 2.7. Cell transfection and co-culture system

For overexpression experiments, astrocytes were transfected with GDNF or NRTN overexpression plasmids or empty vector control using a liposome-based transfection reagent. Transfection efficiency was verified by RT-qPCR and Western blot 48 h after transfection.

For co-culture assays, astrocytes were seeded into the upper chambers of 24-well Transwell plates (0.4 μm pore size), and primary mouse retinal microvascular endothelial cells were seeded into the lower chambers. After 48 h of co-culture, endothelial cells were collected for functional assays.

### 2.8. Endothelial cell function assays

Apoptosis assay: Endothelial cell apoptosis was analyzed by flow cytometry using the Annexin V-FITC/PI kit as described in Section 2.2.

Proliferation assay: Endothelial cell proliferation was detected using an EdU staining kit. Cells were incubated with EdU solution for 2 h, fixed, permeabilized, and stained according to the kit instructions. EdU-positive cells were observed under a fluorescence microscope.

Migration assay: Endothelial cell migration was evaluated using Transwell migration chambers (8 μm pore size). Cells were seeded into the upper chamber, and the lower chamber was filled with complete medium. After 24 h, migrated cells on the lower surface were fixed, stained with crystal violet, and counted under a microscope.

### 2.9. Statistical analysis

All experiments were performed independently in triplicate. Data were expressed as mean ± standard deviation (SD). Statistical analysis was performed using GraphPad Prism software. Comparisons between two groups were performed using Student’s t-test. Multiple group comparisons were conducted using one-way analysis of variance (ANOVA) followed by Tukey’s post hoc test. A value of P < 0.05 was considered statistically significant.

## 3. Results

### 3.1. HG-induces astrocyte senescence and dysfunction

Our previously published study [22] demonstrated that HG conditions reduce astrocyte viability, accelerate cellular senescence, and activate autophagy. Herein, flow cytometry was performed to detect the apoptosis rate of HG-treated astrocytes. Compared with the control group (4.63 ± 0.16), astrocyte apoptosis was significantly increased in the HG group (13.09% ± 0.26%, Fig 1A-B). To determine whether HG triggers inflammatory responses in astrocytes, ELISA was used to measure the secretion of pro-inflammatory mediators COX-2, IL-6, and IL-8 in cell supernatants. Compared with controls, the HG group exhibited significantly elevated levels of COX-2 (from 8.74 ± 0.84 to 19.04 ± 0.55), IL-6 (from 21.38 ± 0.46 to 50.97 ± 0.88), and IL-8 (from 44.73 ± 1.67 to 100.12 ± 1.38), indicating HG-induced astrocytic inflammation (Fig 1C-E). To explore whether blood-retinal barrier (BRB) damage is associated with reduced secretion of glial cell-derived neurotrophic factor (GDNF) and neurturin (NRTN) by senescent astrocytes, RT-qPCR, Western blot, and ELISA were conducted. The data revealed that GDNF protein, mRNA, and secreted levels decreased from 1.00 ± 0.13, 1.00 ± 0.12, and 64.99 ± 0.50 in controls to 0.63 ± 0.09, 0.44 ± 0.03, and 15.15 ± 1.50 in HG-treated astrocytes, respectively, while NRTN correspondingly declined from 1.00 ± 0.14, 1.01 ± 0.12, and 165.35 ± 1.01 to 0.52 ± 0.21, 0.44 ± 0.04, and 37.14 ± 1.50 (Fig 1F-M). Furthermore, RT-qPCR and ELISA revealed significantly increased vascular endothelial growth factor (VEGF) expression in the HG group, with RT-qPCR values rising from 1.002 ± 0.076 to 2.139 ± 0.045 and ELISA values rising from 9.019 ± 0.172 to 26.063 ± 0.246 (Fig 1N-O), suggesting that BRB impairment in diabetic retinopathy (DR) may result from decreased GDNF/NRTN secretion and upregulated VEGF in senescent astrocytes. Our prior animal study [22] identified abnormal expression of the HIF-1α/PPAR-γ/SIRT1 axis in a DR mouse model. Consistently, Western blot and RT-qPCR showed that HIF-1α protein and mRNA levels increased from 1.00 ± 0.06 to 3.09 ± 0.18 and from 1.00 ± 0.07 to 1.91 ± 0.13, respectively, while PPAR-γ protein and mRNA rose from 1.00 ± 0.06 to 1.67 ± 0.19 and from 1.04 ± 0.32 to 2.31 ± 0.52, respectively, and SIRT1 protein and mRNA declined from 1.00 ± 0.15 to 0.50 ± 0.14 and from 1.00 ± 0.02 to 0.49 ± 0.08 in HG-stimulated astrocytes compared with controls (Fig 1P-W), with all differences statistically significant. These findings indicate that HG induces dysregulation of the HIF-1α/PPAR-γ/SIRT1 axis in astrocytes, consistent with in vivo observations.

**Fig 1:**
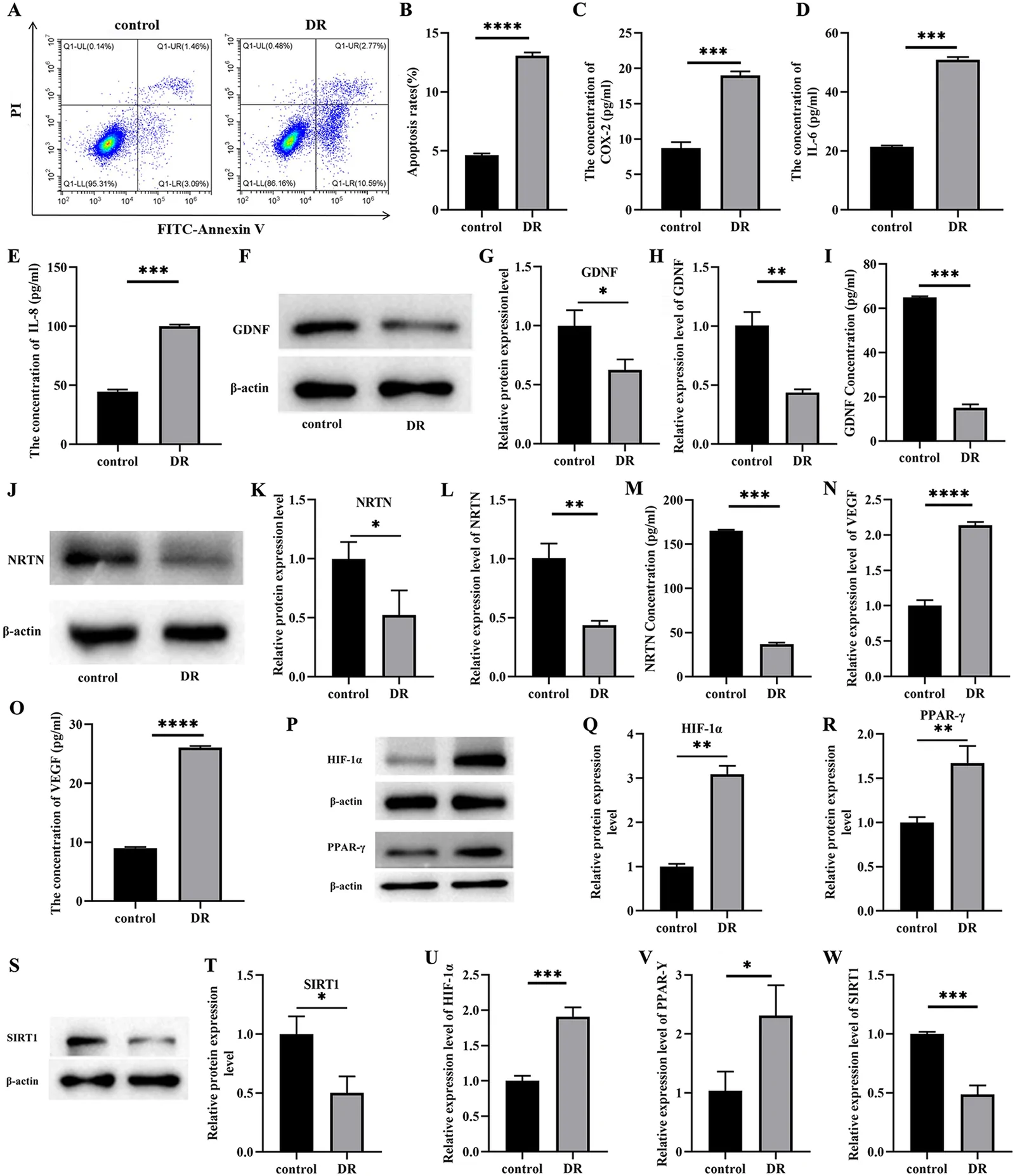
High glucose induces senescence, dysfunction, and HIF-1α/PPAR-γ/SIRT1 axis dysregulation in astrocytes. A-B. The apoptosis rates of astrocytes were detected by flow cytometry. C-E. ELISA was performed to analyze the levels of COX-2, IL-6, and IL-8 in cell supernatants. F-M. The mRNA and protein expression levels of GDNF and NRTN were measured by RT-qPCR and Western blot. N-O. The levels of VEGF were examined. P-W. The mRNA and protein expression levels of HIF-1α, PPAR-γ, and SIRT1 were determined. *P < 0.05, **P < 0.01, ***P < 0.001, ****P < 0.0001. Data are presented as mean ± SD from n = 3 independent biological experiments. Statistical significance was determined by Student’s t-test for comparisons between two groups.

### 3.2. HG mediates astrocytic injury via HIF-1α

Given the upregulated HIF-1α in HG-treated astrocytes, we treated HG-induced senescent astrocytes with the HIF-1α inhibitor PX-478 for 24 h to investigate the regulatory role of HIF-1α in astrocyte senescence. Astrocytes were divided into three groups: control (normal glucose medium), DR (HG medium), and DR + PX-478 (HG medium supplemented with PX-478). Consistent with previous findings [22] that PX-478 alleviates senescence by mitigating cell cycle disruption and excessive autophagy, flow cytometry showed that apoptosis rates increased from 6.14% ± 1.27% in the control group to 15.65% ± 0.67% in the DR group, and were partially reduced to 10.57% ± 0.17% upon PX 478 intervention (Fig 2A-B). ELISA further demonstrated elevated COX-2, IL-6, and IL-8 in the DR group, whereas PX-478 treatment reduced their expression (Fig 2C-E). Specifically, COX-2 levels increased from 7.55 ± 1.00 (pg/ml) in controls to 18.61 ± 0.81 (pg/ml) in the DR group and were partially reduced to 15.04 ± 0.90 (pg/ml) upon PX 478 treatment; IL-6 increased from 21.19 ± 0.70 (pg/ml) to 51.66 ± 0.29 (pg/ml) and decreased to 42.39 ± 1.03 (pg/ml); and IL-8 (pg/ml) increased from 43.30 ± 0.37 (pg/ml) to 103.79 ± 1.49 (pg/ml) and decreased to 73.10 ± 2.07 (pg/ml). These findings suggest that HIF-1α inhibition ameliorates inflammatory responses in senescent astrocytes. To verify the regulatory relationship of the HIF-1α/PPAR-γ/SIRT1 axis in senescent astrocytes, Western blot and RT-qPCR were performed after PX-478 intervention. In DR, PPAR-γ protein and mRNA levels rose from 1.00 ± 0.06 and 1.00 ± 0.07 in controls to 1.53 ± 0.12 and 3.12 ± 0.29, respectively, and were lowered to 1.15 ± 0.12 and 1.08 ± 0.10 by PX-478. Conversely, SIRT1 protein and mRNA dropped from 1.00 ± 0.05 and 1.00 ± 0.08 to 0.57 ± 0.14 and 0.55 ± 0.09, and were restored to 0.87 ± 0.04 and 0.96 ± 0.07 with PX-478 (Fig 2F-K). These results indicate that HIF-1α is positively correlated with PPAR-γ and negatively correlated with SIRT1 in senescent astrocytes. Additionally, HIF-1α inhibition by PX-478 restored the diabetes-induced decline of GDNF and NRTN at both protein and mRNA levels. By WB, GDNF fell from 1.00 ± 0.10 (control) to 0.28 ± 0.17 (DR) and recovered to 0.78 ± 0.11 (DR + PX-478); NRTN followed a similar pattern (1.00 ± 0.08 → 0.38 ± 0.23 → 0.88 ± 0.06). RT-qPCR confirmed these trends for GDNF (1.00 ± 0.11 → 0.38 ± 0.07 → 0.87 ± 0.19) and NRTN (1.02 ± 0.21 → 0.50 ± 0.10 → 1.08 ± 0.13) (Fig 2L-Q), implying that HIF-1α suppression improves endothelial function and attenuates BRB damage in DR.

**Fig 2:**
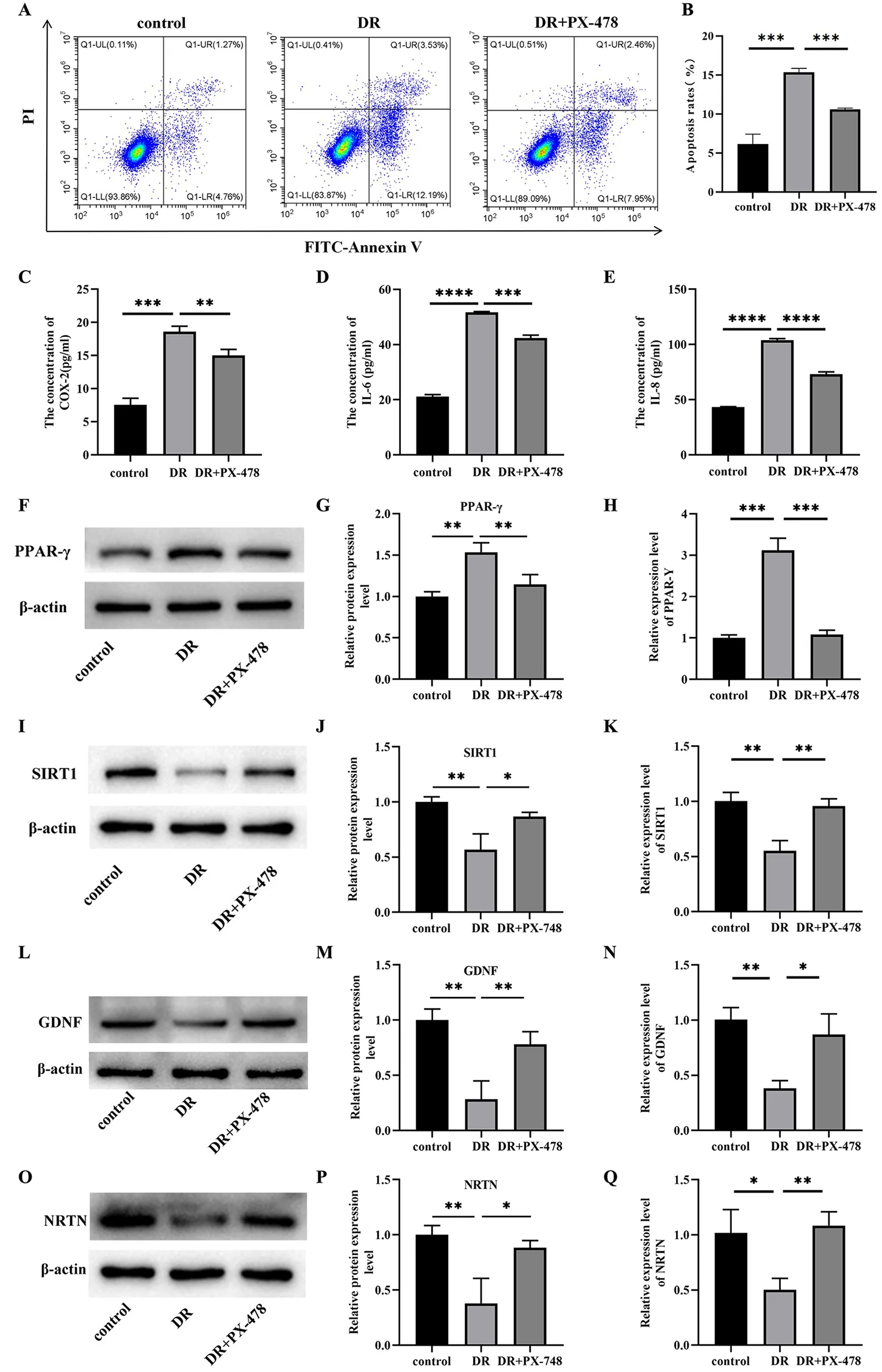
Inhibition of HIF-1α ameliorates high glucose-induced astrocyte injury via regulating the PPAR-γ/SIRT1 pathway and GDNF/NRTN secretion. A-B. Flow cytometry was performed to analyze the apoptosis of astrocytes. C-E. ELISA was used to detect the levels of COX-2, IL-6, and IL-8. F-K. The mRNA and protein expression levels of PPAR-γ and SIRT1 were examined. L-Q. The mRNA and protein expression levels of GDNF and NRTN were examined. *P < 0.05, **P < 0.01, ***P < 0.001, ****P < 0.0001. Data are presented as mean ± SD from n = 3 independent biological experiments. Statistical significance was determined by one-way ANOVA followed by Tukey’s post hoc test for multiple comparisons.

### 3.3. HIF-1α regulates astrocyte senescence via autophagy

To clarify whether HIF-1α regulates astrocyte senescence in an autophagy-dependent manner, HG-treated astrocytes were assigned to three groups: DR, DR + PX-478, and DR + PX-478 + 3-MA (co-treatment with PX-478 and autophagy inhibitor 3-MA). Western blot analysis revealed that, relative to the DR group (Beclin 1: 1.00 ± 0.12; p62: 1.00 ± 0.08; LC3B: 1.00 ± 0.16), the DR + PX-478 group showed increased Beclin 1 (1.98 ± 0.11) and LC3B (2.23 ± 0.07) along with decreased p62 (0.60 ± 0.06), indicative of enhanced autophagy. Co-treatment with 3-MA reversed these changes, as evidenced by reduced Beclin 1 (1.22 ± 0.09) and LC3B (1.24 ± 0.24) and elevated p62 (0.85 ± 0.03), confirming the regulatory role of HIF-1α in autophagy in HG-stimulated astrocytes (Fig 3A-F). Flow cytometry showed that apoptosis was significantly reduced in the DR + PX 478 group compared with the DR group (from 16.56% ± 0.29% to 11.37% ± 0.48%), while co-treatment with 3-MA reversed this anti-apoptotic effect, increasing apoptosis to 14.53% ± 0.18% (Fig 3G-H). Senescence-associated β-galactosidase (SA-β-gal) staining demonstrated alleviated senescence (fewer and weaker-stained cells) in the DR + PX-478 group, which was abrogated by 3-MA co-treatment (Fig 3I). Western blot of the cell cycle regulators p21 and p16 revealed that, compared with the DR group (p21: 1.00 ± 0.02; p16: 1.00 ± 0.07), the DR + PX 478 group showed decreased p21 (0.67 ± 0.01) and p16 (0.60 ± 0.12), indicating improved cell cycle progression and senescence relief. Co-treatment with 3-MA reversed these effects, restoring p21 to 0.94 ± 0.06 and p16 to 0.94 ± 0.04, thereby abrogating the protective effect (Fig 3J-M). Moreover, Western blot analysis revealed that PX 478 treatment significantly increased GDNF and NRTN protein levels in DR-exposed astrocytes, with GDNF rising from 1.00 ± 0.34 in the DR group to 2.34 ± 0.18, and NRTN from 1.00 ± 0.06 to 2.15 ± 0.12. Co-treatment with 3-MA reversed these elevations, reducing GDNF to 1.68 ± 0.12 and NRTN to 1.51 ± 0.21, thereby abolishing the upregulation (Fig 3N-Q). Collectively, these data suggest that HIF-1α inhibition enhances autophagy, mitigates astrocyte senescence, and promotes GDNF/NRTN secretion; such effects are autophagy-dependent.

**Fig 3:**
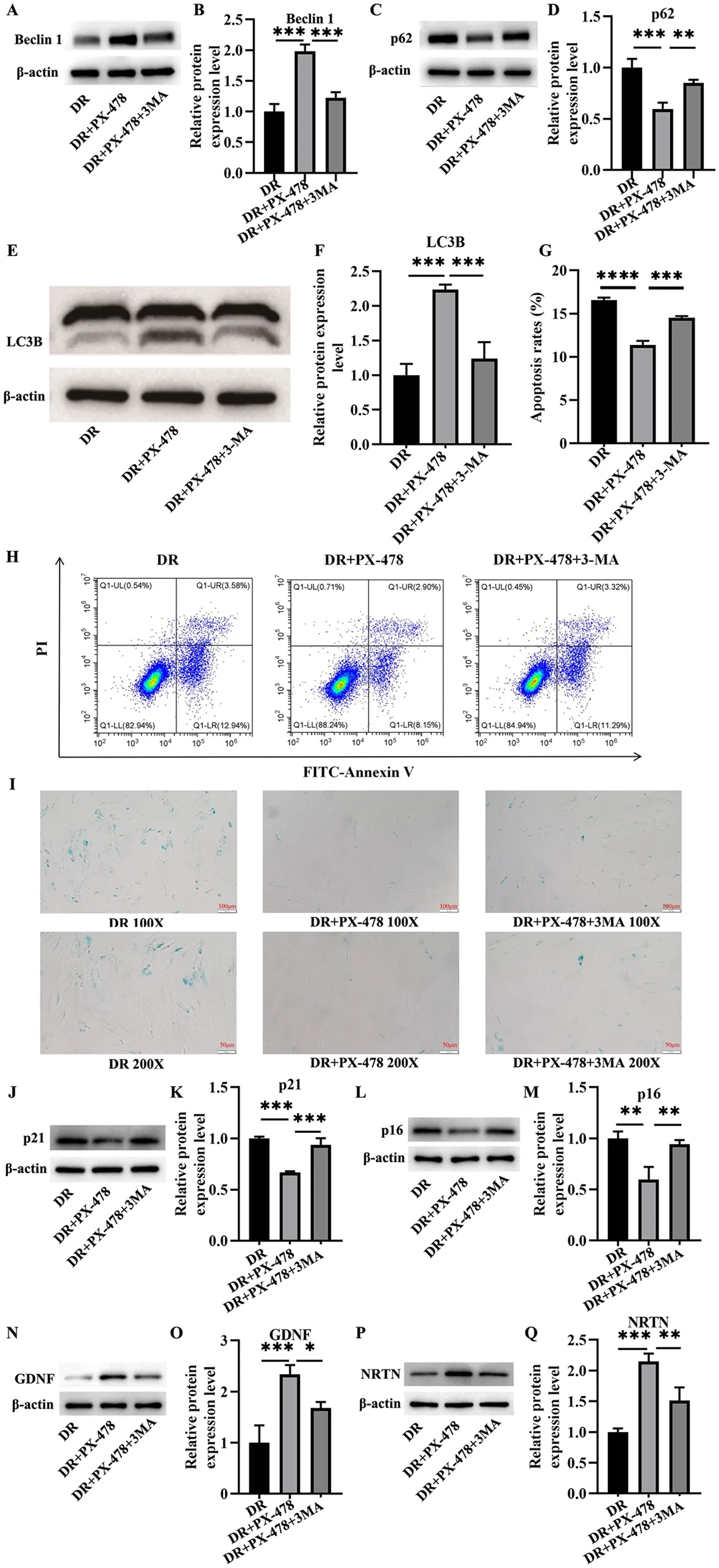
HIF-1α regulates astrocyte senescence and function in an autophagy-dependent manner. A-F. Western blot was conducted to analyze the autophagy-related proteins Beclin 1, p62 and LC3B. G-H. Flow cytometry was used to detect the apoptosis of astrocytes. I. SA-β-gal staining was performed to evaluate cellular senescence. J-M. Western blot analysis was applied to detect the senescence-associated markers p21 and p16. N-Q. The protein expression levels of GDNF and NRTN were examined. *P < 0.05, **P < 0.01, ***P < 0.001, ****P < 0.0001. Data are presented as mean ± SD from n = 3 independent biological experiments. Statistical significance was determined by one-way ANOVA followed by Tukey’s post hoc test for multiple comparisons.

### 3.4. SIRT1 activation attenuates astrocyte senescence via autophagy

We observed elevated HIF-1α and reduced SIRT1 expression in DR retinas [22] and HG-treated astrocytes. We next investigated the role of SIRT1 in astrocyte senescence using three experimental groups: DR, DR + SRT2104 (HG medium with SIRT1 agonist SRT2104), and DR + SRT2104 + 3-MA. Western blot revealed that the DR + SRT2104 group exhibited enhanced LC3B expression (from 1.00 ± 0.06 to 2.36 ± 0.45) and autophagy activation, as evidenced by increased Beclin 1 (from 1.00 ± 0.15 to 1.58 ± 0.09) and decreased p62 (from 1.00 ± 0.05 to 0.58 ± 0.08), whereas co-treatment with 3-MA suppressed autophagy, as shown by reduced LC3B (to 0.90 ± 0.10) and Beclin 1 (to 1.10 ± 0.19) along with elevated p62 (to 0.87 ± 0.04) (Fig 4A-F). SA-β-gal staining showed that SRT2104 alleviated HG-induced astrocyte senescence, while 3-MA reversed this phenotype (Fig 4G). Western blot and RT-qPCR analyses revealed that, compared with the DR group (p21 protein: 1.00 ± 0.06; p21 mRNA: 1.00 ± 0.04; p16 protein: 1.00 ± 0.04; p16 mRNA: 1.00 ± 0.05), the DR + SRT2104 group exhibited marked reductions in p21 (protein: 0.72 ± 0.05; mRNA: 0.58 ± 0.08) and p16 (protein: 0.45 ± 0.14; mRNA: 0.70 ± 0.02) levels. Co-treatment with 3-MA restored both p21 (protein: 0.99 ± 0.16; mRNA: 0.81 ± 0.07) and p16 (protein: 0.80 ± 0.06; mRNA: 0.89 ± 0.05) to levels approaching those of the DR group, indicating that SRT2104-mediated suppression of these senescence markers is autophagy-dependent (Fig 4H-M). Furthermore, SRT2104 significantly increased GDNF and NRTN expression in HG-treated astrocytes, as determined by Western blot and RT-qPCR. Specifically, GDNF protein and mRNA levels rose from 1.00 ± 0.24 and 1.00 ± 0.02 in the DR group to 2.03 ± 0.35 and 2.27 ± 0.04, respectively, while NRTN protein and mRNA increased from 1.00 ± 0.11 and 1.00 ± 0.04 to 2.07 ± 0.17 and 2.15 ± 0.08. Co-treatment with 3-MA reversed these effects, reducing GDNF to 1.35 ± 0.17 (protein) and 1.43 ± 0.09 (mRNA), and NRTN to 1.37 ± 0.24 (protein) and 1.55 ± 0.07 (mRNA), thus blocking the neuroprotective effect (Fig 4N-S). These findings indicate that SIRT1 activation ameliorates HG-induced astrocytic injury in an autophagy-dependent manner.

**Fig 4:**
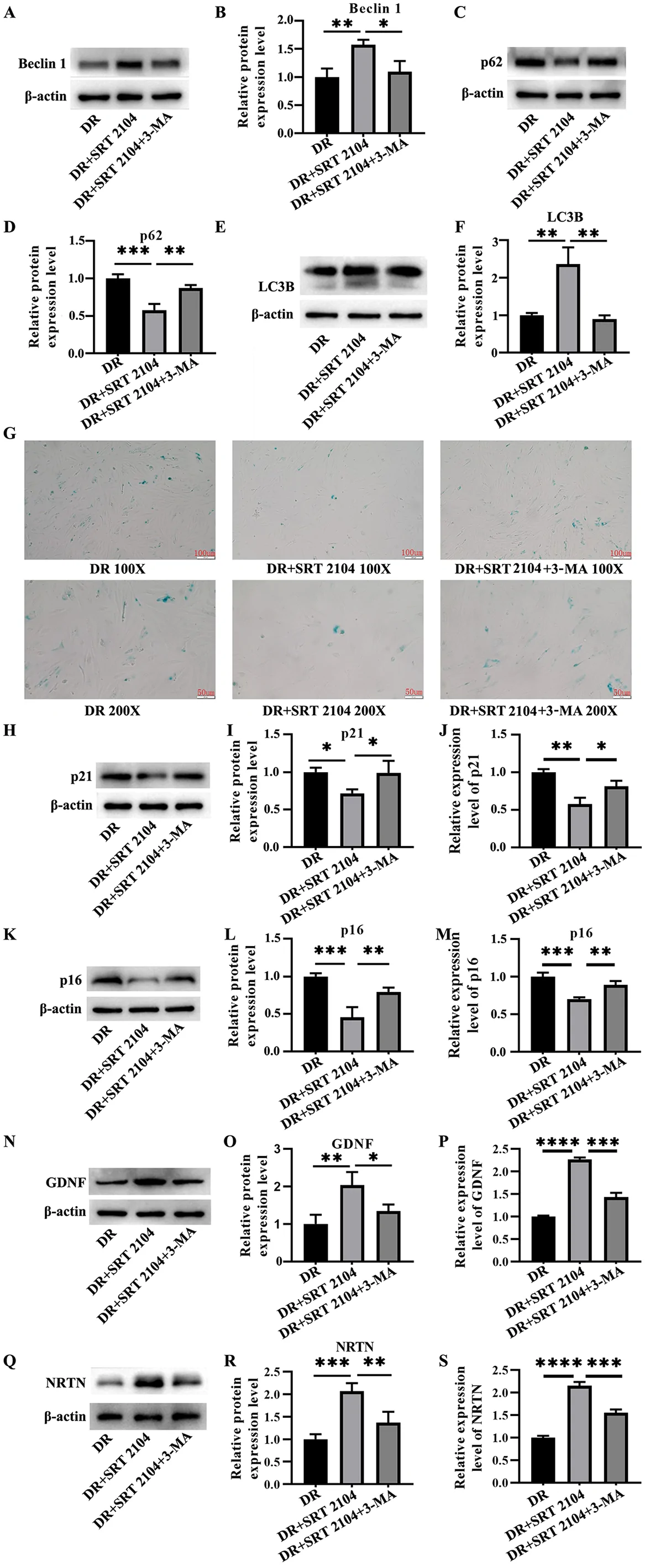
SIRT1 activation attenuates astrocyte senescence and improves function via enhancing autophagy. A-F. Western blot was performed to analyze the expression of Beclin1, p62 and LC3B. G. SA-β-gal staining was used to assess cellular senescence. H-M. The mRNA and protein expression levels of p21 and p16 were detected. N-S. The mRNA and protein expression levels of GDNF and NRTN were determined. *P < 0.05, **P < 0.01, ***P < 0.001, ****P < 0.0001. Data are presented as mean ± SD from n = 3 independent biological experiments. Statistical significance was determined by one-way ANOVA followed by Tukey’s post hoc test for multiple comparisons.

### 3.5. HIF-1α regulates SIRT1 via PPAR-γ

Given the inverse expression patterns of HIF-1α and SIRT1 in DR retinas [22] and HG-treated astrocytes, we further explored whether HIF-1α modulates SIRT1 through PPAR-γ. HG-treated astrocytes were divided into four groups: HG, HG + PX-478, HG + T0070907 (PPAR-γ inhibitor), and HG + PX-478 + T0070907. Western blot and RT-qPCR analyses revealed that, compared with the HG group (protein: 1.00 ± 0.46; mRNA: 1.00 ± 0.06), treatment with PX-478 (protein: 2.30 ± 0.20; mRNA: 2.49 ± 0.24) or T0070907 (protein: 3.49 ± 0.65; mRNA: 5.17 ± 0.42) significantly elevated SIRT1 expression in HG-stimulated astrocytes. However, co-treatment with PX-478 and T0070907 (protein: 3.38 ± 0.52; mRNA: 4.98 ± 0.16) produced no additional increase relative to T0070907 alone, indicating that PPAR-γ inhibition abrogates the regulatory effect of HIF-1α inhibition on SIRT1 (Fig 5A-C). To validate this regulatory axis in normal astrocytes, four groups were established: control, DMOG (HIF-1α agonist), T0070907, and DMOG + T0070907. Western blot and RT-qPCR analyses revealed that DMOG markedly reduced SIRT1 protein and mRNA levels (from 1.00 ± 0.25 to 0.31 ± 0.23 for protein; from 1.00 ± 0.10 to 0.66 ± 0.05 for mRNA), whereas T0070907 alone significantly elevated both protein and mRNA expression (1.62 ± 0.23 and 1.84 ± 0.05, respectively). Notably, co-treatment with DMOG and T0070907 reversed the DMOG-induced downregulation, restoring SIRT1 expression to levels comparable to those of the T0070907 group alone (protein: 1.69 ± 0.23; mRNA: 1.86 ± 0.13), confirming that PPAR-γ inhibition abrogated the suppressive effect of DMOG on SIRT1 (Fig 5D-F). These results collectively confirm that HIF-1α negatively regulates SIRT1 expression partially through PPAR-γ.

**Fig 5:**
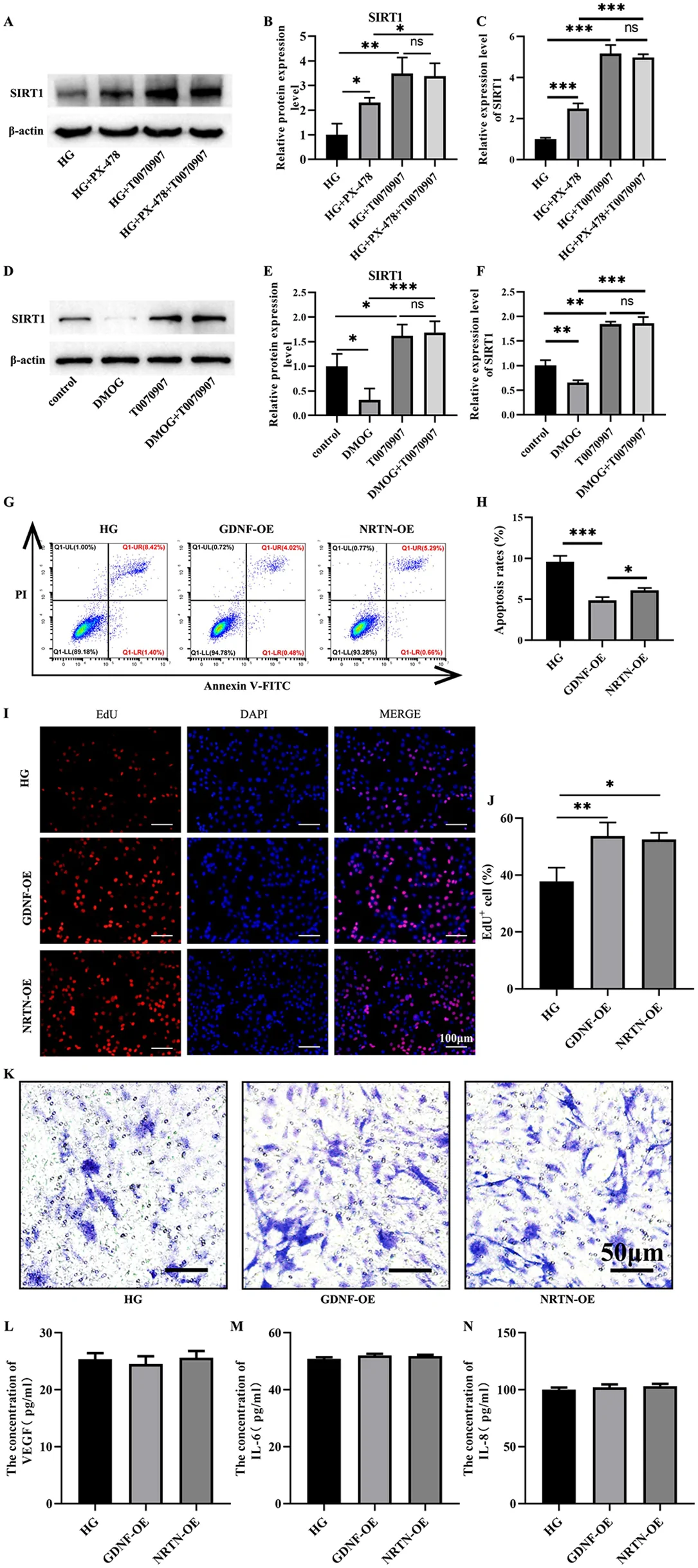
HIF-1α regulates SIRT1 via PPAR-γ, and astrocyte-derived GDNF/NRTN mediates endothelial protection. A-C. Western blot and RT-qPCR were used to detect SIRT1 expression in HG-stimulated astrocytes upon inhibition of HIF-1α and/or PPARγ. D-F. Western blot and RT-qPCR were performed to detect SIRT1 expression in HG-stimulated astrocytes upon HIF-1α activation and/or PPARγ inhibition. G-H. The apoptotic level of retinal endothelial cells was evaluated following co‑cultivation with astrocytes. I-J. The proliferative capacity of endothelial cells was evaluated via EdU assay upon co-culture with astrocytes. K. Migration of endothelial cells detected by Transwell assay after co-culture with astrocytes. L-N. ELISA was used to detect the levels of VEGF, IL-6, and IL-8. *P < 0.05, **P < 0.01, ***P < 0.001. Data are presented as mean ± SD from n = 3 independent biological experiments. Statistical significance was determined by one-way ANOVA followed by Tukey’s post hoc test for multiple comparisons.

### 3.6. Senescent astrocytes impair endothelial function via GDNF/NRTN

Since senescent astrocytes exhibit decreased GDNF and NRTN secretion, we hypothesized that overexpressing GDNF or NRTN in astrocytes could improve retinal microvascular endothelial cell function and BRB integrity under HG conditions. Senescent astrocytes were transfected with GDNF or NRTN overexpression plasmids, followed by Transwell co-culture with retinal microvascular endothelial cells. Flow cytometry showed reduced apoptosis in endothelial cells co-cultured with GDNF- or NRTN-overexpressing astrocytes relative to the HG group, with the apoptotic rates decreasing from 9.58 ± 0.73% in the HG group to 4.87 ± 0.40% in the GDNF-OE group and 6.11 ± 0.27% in the NRTN-OE group (Fig 5G-H). EdU assays revealed improved proliferation of endothelial cells in both overexpression groups, as compared to the HG group (37.81 ± 4.80%), with the percentage of EdU-positive cells increasing to 53.71 ± 4.76% in the GDNF-OE group and 52.53 ± 2.35% in the NRTN-OE group (Fig 5I-J). Transwell migration assays demonstrated enhanced migratory capacity of endothelial cells after co-culture with GDNF- or NRTN-overexpressing astrocytes (Fig 5K). These findings suggest that astrocyte-secreted GDNF and NRTN mitigate HG-induced injury in retinal microvascular endothelial cells. Subsequently, we measured VEGF, IL-6, and IL-8 levels in astrocyte-conditioned medium from HG, GDNF-OE, and NRTN-OE groups. No significant differences were observed among the three groups (Fig 5L-N), indicating that GDNF/NRTN exerts its protective effects via direct paracrine action on endothelial cells.

## 4. Discussion

In the present study, we go beyond our earlier work by elucidating the mechanistic cascade through which HIF-1α controls astrocyte senescence. Unlike the prior focus on phenotypic rescue, we now demonstrate that HIF-1α negatively regulates SIRT1 via PPAR-γ, and that this axis operates through autophagy to modulate the senescence-associated secretory profile, including GDNF and NRTN. Moreover, we provide the first evidence that astrocyte-derived GDNF/NRTN directly protects retinal endothelial cells, revealing a novel neurovascular communication pathway in DR. This study therefore defines a new scientific contribution—the identification of the HIF-1α/PPAR-γ/SIRT1/autophagy/GDNF-NRTN axis—as a potential therapeutic target, which was not addressed in our previous publication.

This study demonstrates that HG induces retinal astrocyte senescence through dysregulation of the HIF-1α/PPAR-γ/SIRT1 signaling axis, which suppresses cytoprotective autophagy. Senescent astrocytes exhibit reduced secretion of GDNF and NRTN, leading to impaired retinal endothelial cell function and BRB disruption. These findings reveal a novel neuroglia-vascular crosstalk mechanism in DR and identify the HIF-1α/PPAR-γ/SIRT1 axis as a potential therapeutic target.

Astrocyte senescence has emerged as a critical driver of early DR pathogenesis [9]. In the diabetic retina, chronic HG exposure triggers oxidative stress, mitochondrial dysfunction, and DNA damage, leading to persistent astrocyte senescence and senescence-associated secretory phenotype activation [27]. Consistent with previous reports [22,28], our study confirmed that HG reduced astrocyte viability, increased apoptosis, upregulated p16 and p21, and elevated SA-β-gal staining. Moreover, HG-treated astrocytes showed increased secretion of pro-inflammatory cytokines including COX-2, IL-6, and IL-8, which further amplify retinal inflammation and vascular injury. These results validate astrocyte senescence and dysfunction as key cellular events in DR. It is worth noting that other retinal glial cell types, particularly Müller glial cells, are also capable of producing GDNF [20,29]. Müller glial cells can upregulate GDNF and its receptors under pathological conditions such as high glucose or light-induced stress [29,30], and GDNF family ligands can trigger signaling pathways in Müller glia [31]. However, the present study primarily focuses on astrocyte–endothelial interactions, as astrocytic endfeet directly enwrap the retinal vessel wall and exert a more direct influence on local BRB regulation. It is possible that astrocytes and Müller glial cells act synergistically to maintain the retinal neurotrophic microenvironment, and this intriguing possibility warrants further investigation.

HIF-1α is a critical sensor of metabolic and hypoxic stress and is strongly implicated in DR progression [32]. Under HG conditions, accumulated reactive oxygen species (ROS) inhibit prolyl hydroxylase activity, leading to HIF-1α stabilization and nuclear translocation [33]. Our study revealed that HIF-1α was significantly upregulated in HG-treated astrocytes. Inhibition of HIF-1α using PX-478 alleviated senescence, apoptosis, and inflammation, indicating a pro-senescent and pro-inflammatory role of HIF-1α in astrocytes. These findings expand the known functions of HIF-1α beyond angiogenesis to the regulation of glial senescence in DR. Extensive *in vitro* and *in vivo* studies have demonstrated the involvement of both HIF-1α and HIF-2α in DR [34,35]. However, these two subunits differ in their retinal cellular distribution and hypoxic responses: HIF-1α is broadly expressed in various retinal cell types, whereas HIF-2α is largely restricted to Müller cells and some endothelial cells; HIF-1α responds rapidly to acute hypoxia, while HIF-2α is more associated with chronic hypoxic adaptation [36].

We further identified PPAR-γ as a critical downstream mediator of HIF-1α. PPAR-γ has been reported to regulate cellular senescence and inflammation in multiple cell types [37,38]. Our results showed that HG increased PPAR-γ expression, while HIF-1α inhibition reduced PPAR-γ levels. More importantly, we demonstrated that HIF-1α negatively regulates SIRT1 through PPAR-γ. Inhibition of either HIF-1α or PPAR-γ restored SIRT1 expression, and simultaneous inhibition of PPAR-γ blocked the effect of HIF-1α inhibition on SIRT1. These data establish a linear HIF-1α/PPAR-γ/SIRT1 signaling cascade in astrocytes.

SIRT1 is a well-known anti-aging molecule that enhances autophagy, reduces oxidative stress, and delays senescence [39,40]. Autophagy is a conserved catabolic process that removes damaged organelles and proteins, maintaining cellular homeostasis and counteracting senescence [41]. Our study showed that HG suppressed autophagy and downregulated SIRT1. Both HIF-1α inhibition and SIRT1 activation restored autophagy activity and alleviated astrocyte senescence. Furthermore, the autophagy inhibitor 3-MA reversed the protective effects of PX-478 and SRT2104, confirming that autophagy is a key downstream effector of the HIF-1α/PPAR-γ/SIRT1 axis in regulating astrocyte senescence.

A major finding of this study is the paracrine crosstalk between senescent astrocytes and retinal endothelial cells. Astrocytes regulate vascular function through secreting neurotrophic factors [42]. We found that HG-induced senescent astrocytes exhibited reduced GDNF and NRTN expression, accompanied by elevated VEGF, a known factor that disrupts BRB integrity [43]. Restoration of GDNF or NRTN in astrocytes significantly improved endothelial cell proliferation and migration, and inhibited apoptosis. These results indicate that loss of GDNF/NRTN-mediated trophic support from senescent astrocytes contributes to endothelial dysfunction in DR. This glia-vascular interaction provides a new mechanistic explanation for BRB breakdown in early DR.

While our findings reveal a regulatory interplay among HIF-1α, PPAR-γ, and SIRT1, interpretation must consider their context-dependent duality—HIF-1α can drive both protective and pathological angiogenesis, whereas SIRT1/PPAR-γ may restrain HIF-1α via PGC-1α or mitochondrial respiration [44,45]. Alternative mechanisms, including SIRT1/AMPK-mediated ERRα activation and Notch signaling modulation, may also contribute [46,47]. Limitations of our in vitro model include the absence of hemodynamic flow, heterotypic cellular crosstalk (neurons, astrocytes, pericytes), systemic metabolic influences, and long-term data. Accordingly, our conclusions remain hypothesis-generating and warrant in vivo validation using genetic/pharmacological animal models, alongside long-term assessments of safety and efficacy, before any translational inference.

Notably, although we verified the protective paracrine effects of GDNF and NRTN on retinal endothelial function, the downstream RET signaling cascade activated by these neurotrophic factors was not explored in the current study. Further investigations focusing on RET phosphorylation and its downstream signaling activation will help fully elucidate the molecular mechanism underlying GDNF/NRTN-mediated endothelial protection in DR, which we consider a valuable direction for future mechanistic research.

## 5. Conclusion

The HIF-1α/PPAR-γ/SIRT1 axis mediates HG-induced retinal astrocyte senescence by inhibiting autophagy. Senescent astrocytes impair retinal endothelial function via reduced GDNF/NRTN secretion. Targeting the HIF-1α/PPAR-γ/SIRT1 axis to restore autophagy and neurotrophic support may represent a potential candidate pathway, although this requires rigorous validation in future in vivo and clinical studies.

## Supporting information

S1 File S1 Data (Figure 1A-1L). Original data for Figure 1A–1L. S2 Data (Figure 1M–1W). Original data for Figure 1M–1W. S3 Data (Figure 2). Original data for Figure 2. S4 Data (Figure 3A–3H). Original data for Figure 3A–3H. S5 Data (Figure 3I). Original data for Figure 3I. S6 Data (Figure 3J–3Q). Original data for Figure 3J–3Q. S7 Data (Figure 4A–4F). Original data for Figure 4A–4F. S8 Data (Figure 4G). Original data for Figure 4G. S9 Data (Figure 4H–4S). Original data for Figure 4H–4S. S10 Data (Figure 5). Original data for Figure 5.
(DOCX)
