# Supplementary material for: HIF-1α/PPAR-γ/SIRT1 axis drives astrocyte senescence and regulates endothelial function via GDNF/NRTN in diabetic retinopathy
Source: PLoS One. 2026 Nov 6;21(11):e0362127. doi: 10.1371/journal.pone.0362127 (PMC13742964; doi:10.1371/journal.pone.0362127)
Supplement: Supplementary file 1 [file pone.0362127.s001.docx]

1F-GDNF

1F-β-actin

1J-NRTN

1F-β-actin

1P-HIF-1α

1P-β-actin

1P-PPAR-γ

1P-β-actin

1S-SIRT1

1S-β-actin

2F-PPAR-γ

2F-β-actin

2I-SIRT1

2I-β-actin

2L-GDNF

2L-β-actin

2O-NRTN

2O-β-actin

3A-Beclin 1

3A-β-actin

3C-p62

3C-β-actin

3E-LC3B

3E-β-actin

3J-p21

3J-β-actin

3L-p16

3L-β-actin

3N-GDNF

3N-β-actin

3P-NRTN

3P-β-actin

4A-Beclin 1

4A-β-actin

4C-p62

4C-β-actin

4E-LC3B

4E-β-actin

4H-p21

4H-β-actin

4K-p16

4K-β-actin

4N-GDNF

4N-β-actin

4Q-NRTN

4Q-β-actin

5A-SIRT1

5A-β-actin

5D-SIRT1

5D-β-actin
